# The role of acrolein for E-cigarette vapour condensate mediated activation of NADPH oxidase in cultured endothelial cells and macrophages

**DOI:** 10.1007/s00424-023-02825-9

**Published:** 2023-06-07

**Authors:** Ivana Kuntic, Marin Kuntic, Matthias Oelze, Paul Stamm, Angelica Karpi, Hartmut Kleinert, Omar Hahad, Thomas Münzel, Andreas Daiber

**Affiliations:** 1 Department for Cardiology 1, University Medical Center Mainz, Molecular Cardiology, Geb. 605, Langenbeckstr. 1, 55131 Mainz, Germany; 2 DZHK (German Center for Cardiovascular Research), Partner Site Rhine-Main, Mainz, Germany; 3 Department of Pharmacology, University Medical Center, Mainz, Germany

**Keywords:** E-cigarettes, Acrolein, Endothelial cells, Macrophages, Oxidative stress

## Abstract

Electronic cigarettes (E-cigarettes) have recently become a popular alternative to traditional tobacco cigarettes. Despite being marketed as a healthier alternative, increasing evidence shows that E-cigarette vapour could cause adverse health effects. It has been postulated that degradation products of E-cigarette liquid, mainly reactive aldehydes, are responsible for those effects. Previously, we have demonstrated that E-cigarette vapour exposure causes oxidative stress, inflammation, apoptosis, endothelial dysfunction and hypertension by activating NADPH oxidase in a mouse model. To better understand oxidative stress mechanisms, we have exposed cultured endothelial cells and macrophages to condensed E-cigarette vapour (E-cigarette condensate) and acrolein. In both endothelial cells (EA.hy 926) and macrophages (RAW 264.7), we have observed that E-cigarette condensate incubation causes cell death. Since recent studies have shown that among toxic aldehydes found in E-cigarette vapour, acrolein plays a prominent role, we have incubated the same cell lines with increasing concentrations of acrolein. Upon incubation with acrolein, a translocation of Rac1 to the plasma membrane has been observed, accompanied by an increase in oxidative stress. Whereas reactive oxygen species (ROS) formation by acrolein in cultured endothelial cells was mainly intracellular, the release of ROS in cultured macrophages was both intra- and extracellular. Our data also demonstrate that acrolein activates the nuclear factor erythroid 2-related factor 2 (Nrf2) antioxidant pathway and, in general, could mediate E-cigarette vapour-induced oxidative stress and cell death. More mechanistic insight is needed to clarify the toxicity associated with E-cigarette consumption and the possible adverse effects on human health.

## Introduction

Electronic cigarettes (E-cigarettes) were introduced to the market about 20 years ago and have gained immense popularity. Even though they have been marketed as a method for smoking cessation, over half of the adults over 18 currently using E-cigarettes report that they have never smoked tobacco cigarettes before (according to the Truth Initiative 2021 [79]). Additionally, there is a lack of strict regulation regarding E-cigarettes, especially since they are supposed to be used as a smoking cessation device [5]. Most of the studies that looked at the health effects of E-cigarettes are oriented towards comparing tobacco smoking and vaping, reporting that vaping is less detrimental than smoking tobacco cigarettes [11, 26, 30]. Adverse effects of tobacco cigarettes are usually attributed to the vast number of chemicals found in burned tobacco leaves and smoke, as well as nicotine, which is known to impact cardiovascular and endocrine systems [77, 82]. Even though E-cigarette liquid contains only a handful of chemicals, reports point to E-cigarettes causing impairment in vascular and cardiac function [18, 52, 76]. Some studies have observed increased vascular inflammation and oxidative stress when using E-cigarette liquids without nicotine or added aroma [10, 41, 59]. So far, there is a lack of mechanistic insight into these E-cigarette health effects, and further investigations are warranted.

The inner lining of blood vessels, which consists of a monolayer of endothelial cells, is an essential mediator of vascular function and plays an active role in the vasoconstriction-vasodilation balance of the vessels, thrombosis, inflammation and immune response [15, 73]. Impairment of these functions could lead to hypertension, atherosclerosis and inflammatory diseases [67, 73]. During atherosclerosis onset, immune cells infiltrate the endothelium and mature into macrophages. Macrophages propagate the inflammatory response and produce local oxidative stress [19]. We have previously established that NOX2 (gp91phox subunit of the nicotinamide adenine dinucleotide phosphate (NADPH) oxidase) knockout mice were protected against E-cigarette-induced vascular dysfunction [41]. Since NOX2 is the phagocytic NADPH oxidase isoform, our previous results suggest that immune cell activation may play a role in the detrimental cardiovascular effects of E-cigarette vaping, also by causing endothelial dysfunction. NADPH oxidase activation can be mediated through many factors, such as growth factors (platelet-derived growth factor—PDGF and epidermal growth factor—EGF), cytokines (tumour necrosis factor—TNF and interleukin 1—IL-1), endothelin-1 and angiotensin II (which act through a G-protein coupled receptor pathway) [28, 58]. The cellular redox status also regulates NADPH oxidase by inhibiting a Rac1-dependent feedback loop or activating an H_2_O_2_-dependent feed-forward loop [38]. As cytosolic subunits like Rac1 translocate to the membrane when the NADPH oxidase complex is activated, it is possible to evaluate the activation of the complex by determining the concentration of those subunits in membrane fractions of the cell lysates [54].

E-cigarette liquid base usually consists of propylene glycol and glycerine, known as non-toxic [3, 57]. However, upon heating, propylene glycol and glycerine undergo a chemical transformation and produce aldehydes and other products [3, 9]. Our group already established that acrolein is potentially the most toxic reactive aldehyde from glycerine heating [41]. Acrolein is an electrophilic compound that reacts with nucleophilic side chain residues of amino acids such as cysteine, lysine and histidine [8]. Due to this highly reactive nature, acrolein is a potential activator of the NADPH oxidase complex via electrophilic regulation of thiol groups involved in the active multi-protein-complex formation.

Acrolein was also shown to activate the nuclear factor erythroid 2-related factor 2 (Nrf2) transcription factor [75]. Nrf2 is inactive in the cytosol, where it is bound to Keap1, and in the presence of oxidative stress or electrophiles, it separates from Keap1 and translocates into the cell nucleus [20]. This translocation of the Nrf2 transcription factor increases the upregulation of antioxidant defence enzymes such as HO-1 and NQO-1. Acrolein can interact with Nrf2 directly or deplete glutathione (GSH), a redox trigger for Nrf2 activation [42]. Our previous study in mice also confirmed this, where aortic HO-1 protein expression was significantly increased upon exposure to E-cigarette vapour [41].

Here, we evaluated acrolein-dependent E-cigarette vapour effects on cultured endothelial and immune cells by determining cell viability, general oxidative stress induction, NADPH oxidase complex activation and Nrf2-dependent antioxidant mechanisms.

## Materials and methods

### E-cigarette liquid and E-cigarette vapour condensate

**Fig. 1:**
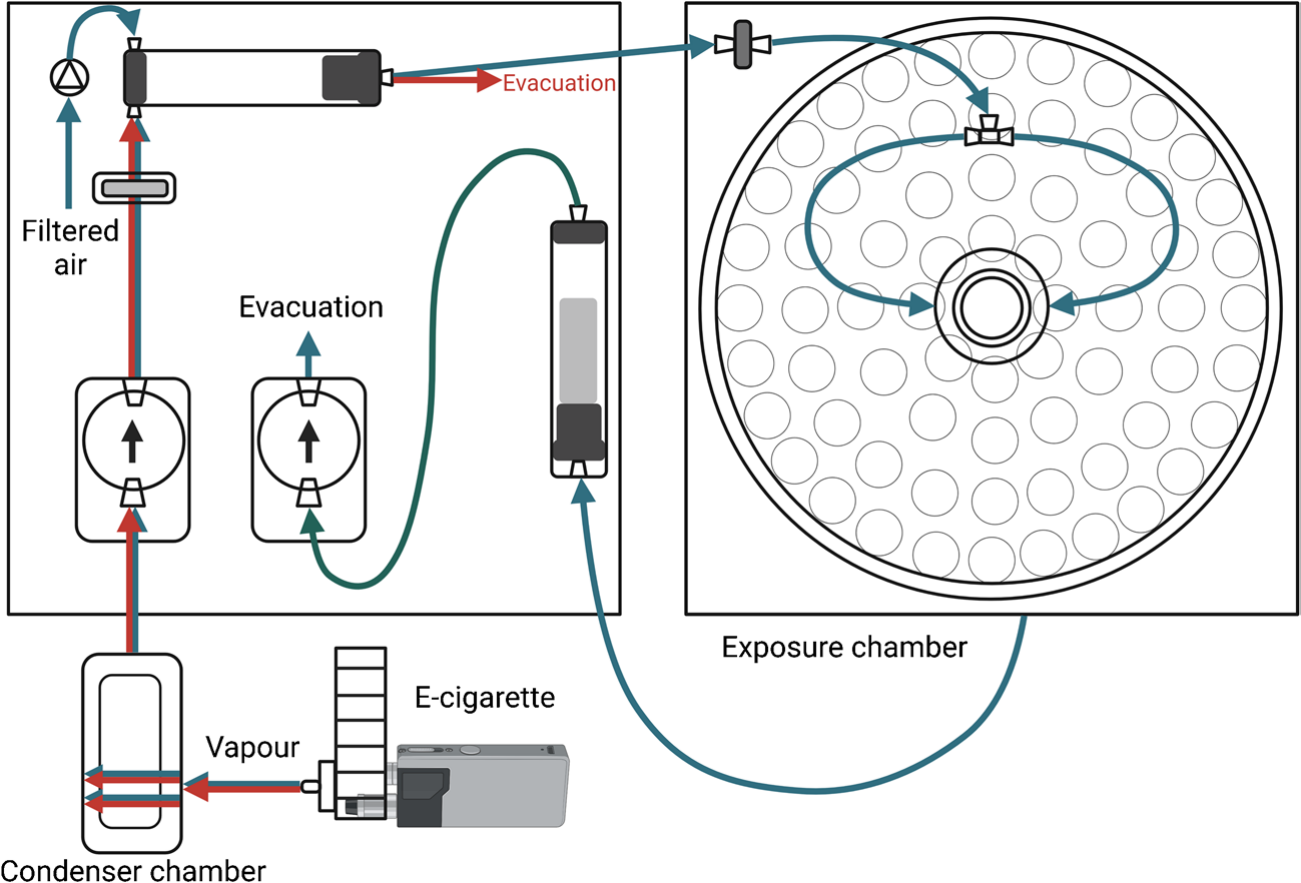
E-cigarette exposure system scheme. Blue arrows show standard airflow when the exposure chamber is connected. Red arrows show airflow when the exposure chamber is disconnected, the way it was used for condensing E-cigarette vapour in this paper. The condensate was collected from the condenser chamber. The exposure system is operated by the flexiWare 8.0 software (Emka Technologies, France). Created with BioRender.com

E-cigarette liquid was obtained from German Flavours (Sprockhövel, Germany). The liquid is a 1:1 mixture of 1,2-propylene glycol and glycerine without added aromas or nicotine. The liquid was heated with an E-cigarette device (eVic-VTC Mini, Joyetech, Shenzhen, China) custom designed to fit an E-cigarette exposure system (InExpose, Emka Technologies, France) shown in Fig. 1 and described in detail in our previous work [41]. The vapour was produced every 30 s for 3 s (puff volume 55 mL), then condensed to a liquid and used for further experiments. The rate of E-cigarette vapour condensate production was around 300 µL/h. The collected condensate was kept at 4 °C in the dark for up to 24 h before cell exposure and warmed to 37 °C right before mixing with the cell medium and using on the cells.

### Cell culture

EA.hy 926 cells (immortalized human endothelial cells), DLD1-HO1-prom cell lines and RAW 264.7 immortalized murine macrophages were used in the experiments. EA.hy cell line was a kind gift by C.-J. S. Edgell (the University of North Carolina, Chapel Hill, USA) [21]. RAW 264.7 cell macrophages were obtained from LGC Standards (Wesel, Germany). DLD1-HO1-prom cell line is a human DLD-1 cell line transfected with a human HO-1 promoter region attached to a luciferase reporter gene (continuous culture at the Department of Pharmacology, University Medical Center Mainz, Germany). EA.hy 926 and RAW 264.7 cells were seeded in 6-well plates in Dulbecco’s modified Eagle’s medium (DMEM, Sigma-Aldrich) with 10% foetal calf serum, 2 mM L-glutamine, 1 mM sodium pyruvate, 100 IU/mL penicillin and 100 μg/mL streptomycin as previously described [36, 71]. DLD1-HO1-prom cells were seeded in 96-well plates in DMEM with 10% foetal calf serum, 1% penicillin and streptomycin, and 2 mM pyruvate. Cells were kept at 37 °C and 10% CO_2_ until they were 70–80% confluent, as previously described [66]. For the experiment, they were incubated with either 1% DMSO (control) or increasing concentrations of acrolein (0.33, 1, 3.3, 10, 33 µM; these concentrations were estimated to match concentrations in the E-cigarette vapour condensate [41]) or E-cigarette vapour condensate (1:150, 1:100, 1:75, 1:50, 1:25; ratio of condensate to the cell medium). All solutions of acrolein (Sigma-Aldrich, St. Louis, USA) or E-cigarette vapour condensate were prepared in 1% DMSO. Cells were incubated for 3 days (EA.hy 926 and RAW 264.7 cells) or 2 days (DLD1-HO1-prom cells). The exposure medium with acrolein or E-cigarette vapour condensate was changed every 24 h. Treatments were heated up to 37 °C, added to the medium, mixed well and then added to the cells under sterile conditions. Microscopic images of the wells were taken each day while changing the exposure medium.

### Cell viability

The viability of the EA.hy 926 cells was determined by counting adherent cells using light microscopy at 20 × zoom. Cells were counted at 24 h, 48 h and 72 h of the treatment during medium change, and the old medium was collected each day and used for LDH assay. The viability of the RAW 264.7 cells was determined using a haemocytometer on the last day of exposure and supplemented by images of adherent cells using light microscopy.

### Lactate dehydrogenase (LDH) assay

Cytotoxicity Detection Kit (LDH Kit Plus, Roche, Basel, Switzerland) was used to determine cell death in EA.hy 926 cells, as described by the manufacturer. Cell medium was collected every 24 h of the treatment from each well and frozen at − 80 °C. Diluted cell medium (1:5) was mixed in a 96-well plate with LDH detection mix and incubated for 30 min in the dark. Stop solution was added, the plate was shaken for 10 s, and the absorbance was measured at 490 nm using a Mithras^2^ plate reader (Berthold Technologies, Bad Wildbad, Germany). In RAW 264.7 cells, the assay did not produce reliable results (data not shown).

### Oxidative burst

Oxidative burst was measured from cell suspension of RAW 264.7 macrophages in analogy to the previously described burst measurement in whole blood or isolated human granulocytes [14]. Samples were diluted into matching cell numbers (10^6^ cells/mL) using PBS/Ca^2+^/Mg^2+^ (1 mM). The reaction was induced using zymosan A (50 µg/mL, Sigma-Aldrich), and the luminescence of the chemiluminescent probe L-012 (100 µM, Wako Pure Chemical Industries, Osaka, Japan) was measured using Mithras^2^ plate reader (Berthold Technologies, Bad Wildbad, Germany) every 5 min for a total time of 60 min. Oxidative burst was done only in RAW 264.7 macrophages and not in the EA.hy 926 endothelial cells as only phagocytic cells are capable of producing substantial oxidative burst [74].

### Total oxidative stress

Dihydroethidium (DHE)-dependent fluorescence microtopography was used to assess oxidative stress in both EA.hy 926 and RAW 264.7 cell cultures as described [16, 41]. Plates containing cell cultures were washed two times with pre-warmed PBS, then incubated with 1 µM DHE (in PBS) for 30 min at 37 °C. After incubation, cells were rewashed with PBS and imaged under a fluorescence microscope (Axiovert 40CFL with Axiocam MRm, Zeiss, Germany). The excitation wavelength was set at 510–520 nm, while the emission wavelength was 580–610 nm. Images were quantified using the GelPro software. After imaging, cells were additionally incubated with 25 µM DHE (in PBS) for 30 min at 37 °C. The PBS fraction was collected and used to determine DHE oxidation by extracellular superoxide and H_2_O_2_ via HPLC-based fluorescence detection of 2-hydroxyethidium (2-HE) and ethidium (E +), the respective specific and unspecific oxidation products of DHE. Cells were then lysed with a 1:1 solution of acetonitrile and water, and the lysate was used to determine the intracellular oxidation products of DHE as described [35, 39, 81]. HPLC system (Jasco, Japan) with fluorescent detection used a C18-Nucleosil 100–3 (125 × 4) column (Macherey and Nagel). A gradient of 90% acetonitrile/10% water solvent and 50 mM citrate buffer (pH 2.2) was used as a mobile phase, with percentages of organic solvent as follows: 0 min—36%, 7 min—40%, 8 min—95%, and 13 min—36%. The flow was 1 mL/min, with DHE detection at 355 nm (absorbance), and 2-HE and E + detection at 480 nm excitation and 580 nm emission (fluorescence). The retention time of 2-HE and E + was around 3.2 min and 3.7 min, respectively, and the signal was compared to a 1 µM standard.

### Western blot

Cell protein samples were analysed using a standard Western blot technique described in [40, 41]. Protein samples were stained with 3 × Laemmli buffer and ran on a 10% loading gel and 4% stacking gel, at 130 V. Depending on the concentration of the samples, 10-well or 15-well gels were used so that the total protein amount was at least 20 µg per well. After separation, proteins were transferred to the nitrocellulose membrane at 80 mA and 4 °C overnight. Membranes were blocked with blocking buffer (3% bovine serum albumin in TBST) for 1 h. Afterward, the membranes were incubated with primary antibody for Rac1 (BD Biosciences, USA, mouse monoclonal antibody, 1:1000 dilution) overnight (about 15 h) at 4 °C. Alpha-actinin (Sigma-Aldrich, mouse monoclonal, 1:2500 dilution) was used as housekeeping protein for normalisation of loading and transfer. After washing the membranes with washing buffer (TBST), the membranes were incubated with peroxidase-coupled secondary antibody (anti-mouse, Vektor, 1:10,000 dilution) for 2 h at room temperature. The membrane was visualized with an enhanced chemiluminescence kit (SuperSignal ECL kit, Thermo Scientific) and detected by ChemiLux Imager (Intas, Göttingen, Germany) and quantified using the GelPro software.

### Luciferase-dependent heme-oxygenase-1 promoter activity reporter assay

DLD1-HO1-prom cells were used to assess the promoter activity of heme-oxygenase-1 in an Nrf2-dependent fashion after acrolein exposure. After a 2-day treatment with acrolein, the cells were lysed using Passive Lysis Buffer (Promega, USA) for 30 min at 37 °C. The lysate was treated with a luciferase assay solution (d-luciferin 12.5 µM, coenzyme A 27 mM, ATP 100 mM, Tricin 0.5 M, MgSO_4_ 1 M, EDTA 0.5 M, DTT 1 M). The chemiluminescence was measured with a 10 s acquisition time on Mithras^2^ plate reader (Berthold Technologies, Bad Wildbad, Germany). The detailed protocol of the assay was previously published [66].

### Statistical analysis

GraphPad Prism 9 was used for the statistical analysis of all the data and visualization of all the graphs. One-way ANOVA (with Tukey post hoc test for comparison of multiple means) or, where appropriate, equivalent non-parametric Kruskal–Wallis test (Dunn multiple comparisons) was used as specified in the figure legends. Two-way ANOVA (with Tukey post hoc test) or, when applicable, a mixed-effects model (with Tukey post hoc test) was used for the EA.hy cell viability as specified in the figure legends. A two-tailed *t*-test was used for comparison of the HPLC results. When analysed, data are presented as mean ± range as a jitter plot, and a *p*-value ≤ 0.05 is considered statistically significant. Standard deviation (SD) was used for the kinetic traces of oxidative burst.

## Results

### Effects of acrolein and E-cigarette condensate on viability of cultured human endothelial cells

**Fig. 2:**
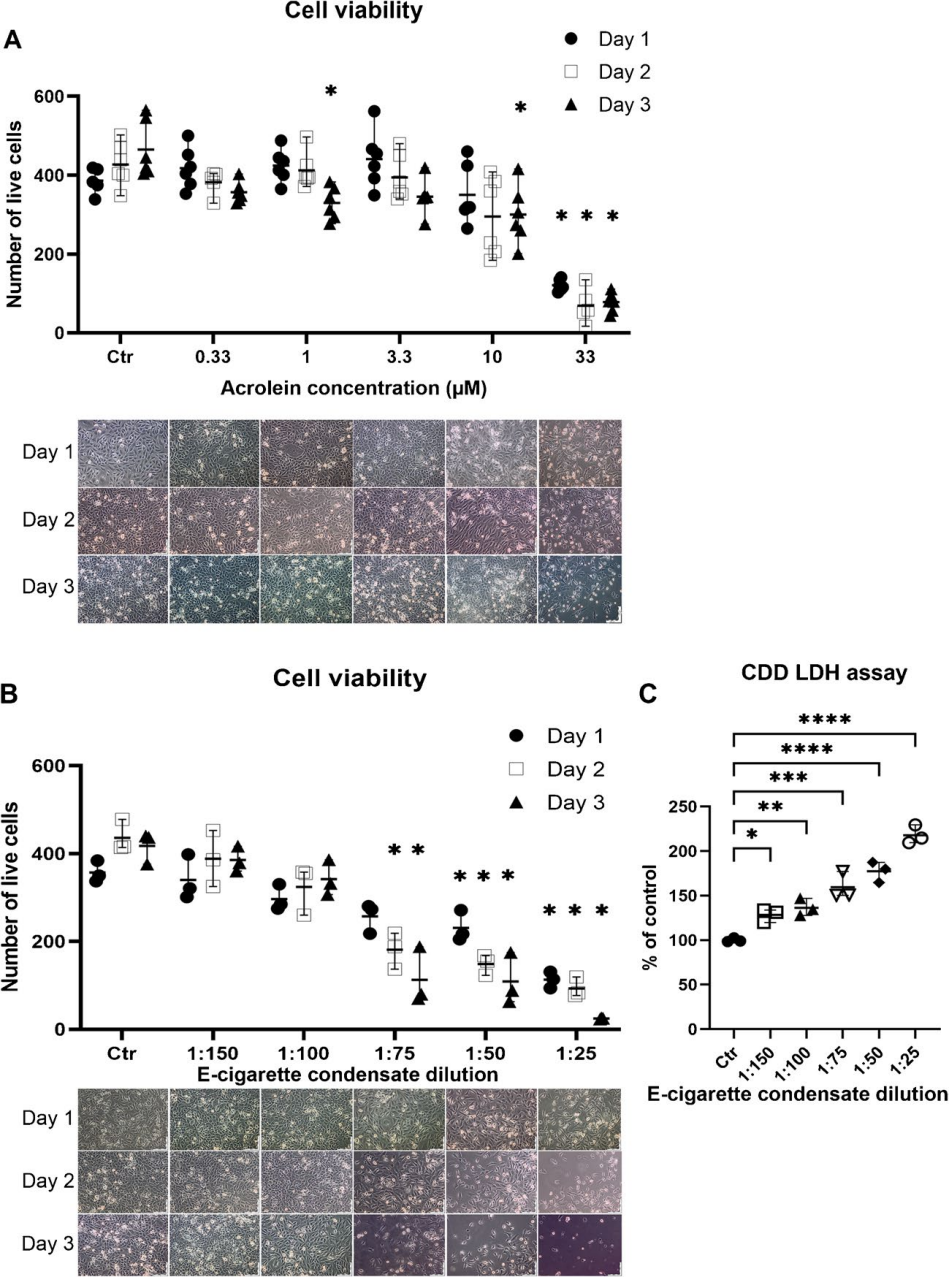
Effects of acrolein and E-cigarette condensate on EA.hy 926 cells. EA.hy 926 cells were incubated for 3 days with solvent (1% DMSO, named control) or **A** different concentrations of acrolein in DMSO (0.33, 1, 3.3, 10, 33 µM) or **B** E-cigarette condensate (1:150, 1:100, 1:75, 1:50, 1:25). Pictures were taken each day to observe the decrease in cell viability. The viable cells were counted from the pictures as the cells with a visible nucleus. The data are shown as mean ± range from *n* = 3–6 independent experiments. By recommendation of the statistical package from the Prism 9.0 software, a mixed-effects model (due to missing matched values) was used for data in panel **A**, whereas conventional 2-way ANOVA was used for data in panel **B**. Significance is indicated when *p* < 0.05 between the treated and control groups. **C** Cell death assay (lactate dehydrogenase [LDH]-based) was performed on the collected cell medium after the 3-day exposure to confirm the manual cell counts from images presented in panels **A** and **B**. The data are shown as mean ± range from *n* = 3 independent experiments. Conventional 1-way ANOVA analysis was performed. Significance is indicated as *, **, *** and **** when *p* < 0.05, *p* < 0.01, *p* < 0.001 and *p* < 0.0001 respectively

Treating human endothelial (EA.hy 926) cells with acrolein caused a concentration and time-dependent loss of viability (Fig. 2A). The highest concentration of acrolein used in the experiment (33 µM) had a statistically significant impact on the viability of the cells from the first day onward. Statistically significant loss of viability for 1 and 10 µM acrolein concentrations was observed after the third exposure day, but other concentrations showed a similar trend.

EA.hy 926 cells treated with E-cigarette vapour condensate also expressed concentration and time-dependent loss of viability (Fig. 2B). Unlike with acrolein treatment, E-cigarette vapour condensate treatment showed a statistically significant loss of viability even on the second day with all treatment concentrations except for the two lowest (1:150 and 1:100). The highest concentration of E-cigarette vapour condensate used in the experiment (1:25) had a statistically significant impact on the viability of the cells from the first day onward. The same effect was noted with cell death detection assay (CDD) after 3-day exposure (Fig. 2C).

### Effects of acrolein and E-cigarette vapour on viability and extracellular ROS formation of cultured macrophages

**Fig. 3:**
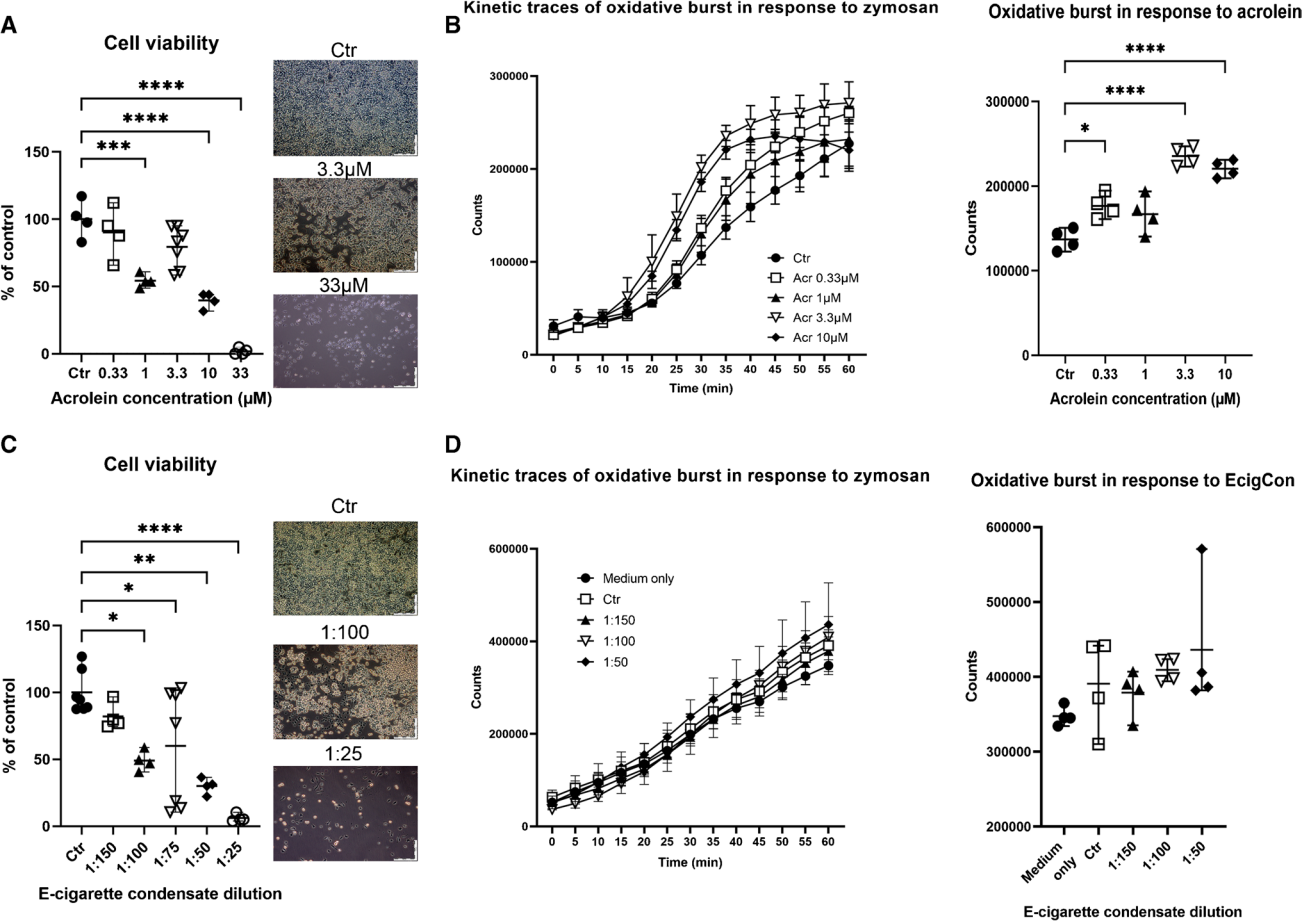
Effects of acrolein and E-cigarette condensate on RAW 264.7 macrophages. RAW 264.7 macrophages were incubated for 3 days with solvent (1% DMSO, named control) or different concentrations of acrolein in DMSO (0.33, 1, 3.3, 10, 33 µM) (**A**) or E-cigarette condensate (1:150, 1:100, 1:75, 1:50, 1:25) (**C**). Pictures were taken each day as representative images. The viable cells were counted using a haemocytometer. The data are shown as mean ± range from *n* = 4 independent experiments. **B** The oxidative burst in whole cells after acrolein incubation is shown both with full kinetic traces and at the time point when traces hit a plateau (35 min). Data at the 35-min point are shown as mean ± range from *n* = 4 pooled samples. **D** The oxidative burst in whole cells after E-cigarette condensate incubation is shown both with full kinetic traces and at the last time point (60 min) as mean ± range from *n* = 4 independent experiments. Error bars in the kinetic traces in panels **B** and **D** represent SD. Conventional 1-way ANOVA analysis was performed for all jitter plots. Significance is indicated as *, **, *** and **** when *p* < 0.05, *p* < 0.01, *p* < 0.001 and *p* < 0.0001 respectively

Treatment of the macrophages (RAW 264.7) with acrolein also caused a concentration-dependent loss of cell viability (Fig. 3A). Since macrophages tend to overlap when cultivated and the nuclei cannot be effectively seen, cell counting was performed using a haemocytometer only after the third exposure day. The highest concentration of acrolein used in the experiment (33 µM) significantly impacted cell viability that the cells could not be easily in further experiments. Stimulation of oxidative burst was performed after the third exposure day, and kinetic traces show an increase in macrophage-derived reactive oxygen species (ROS) formation in treated cells (Fig. 3B), most likely measured in the extracellular compartment by the burst assay. When the results were compared at the 35-min mark (when kinetic traces reached a plateau), a statistically significant increase in the oxidative burst was observed in the cells treated with 0.33, 3.3 and 10 µM acrolein compared to the control.

RAW 264.7 macrophages treated with E-cigarette vapour condensate again showed a concentration-dependent loss of cell viability (Fig. 3C). Cell counting was again performed using a haemocytometer after the third exposure day. Stimulation of oxidative burst was performed after the third exposure day, and kinetic traces show an increase in macrophage-derived extracellular ROS formation in treated cells (Fig. 3D). The E-cigarette vapour condensate concentration of 1:25 was not used for oxidative burst measurements, as the cell viability was not high enough. When the results were compared at the 60-min mark (the time point when kinetic traces hit the highest point; this time no plateau was perceived), no statistically significant increase in the oxidative burst was detected in the cells treated with E-cigarette vapour condensate compared to the control.

### Acrolein effects on intracellular ROS formation and membrane Rac1 protein content in EA.hy and RAW cells

**Fig. 4:**
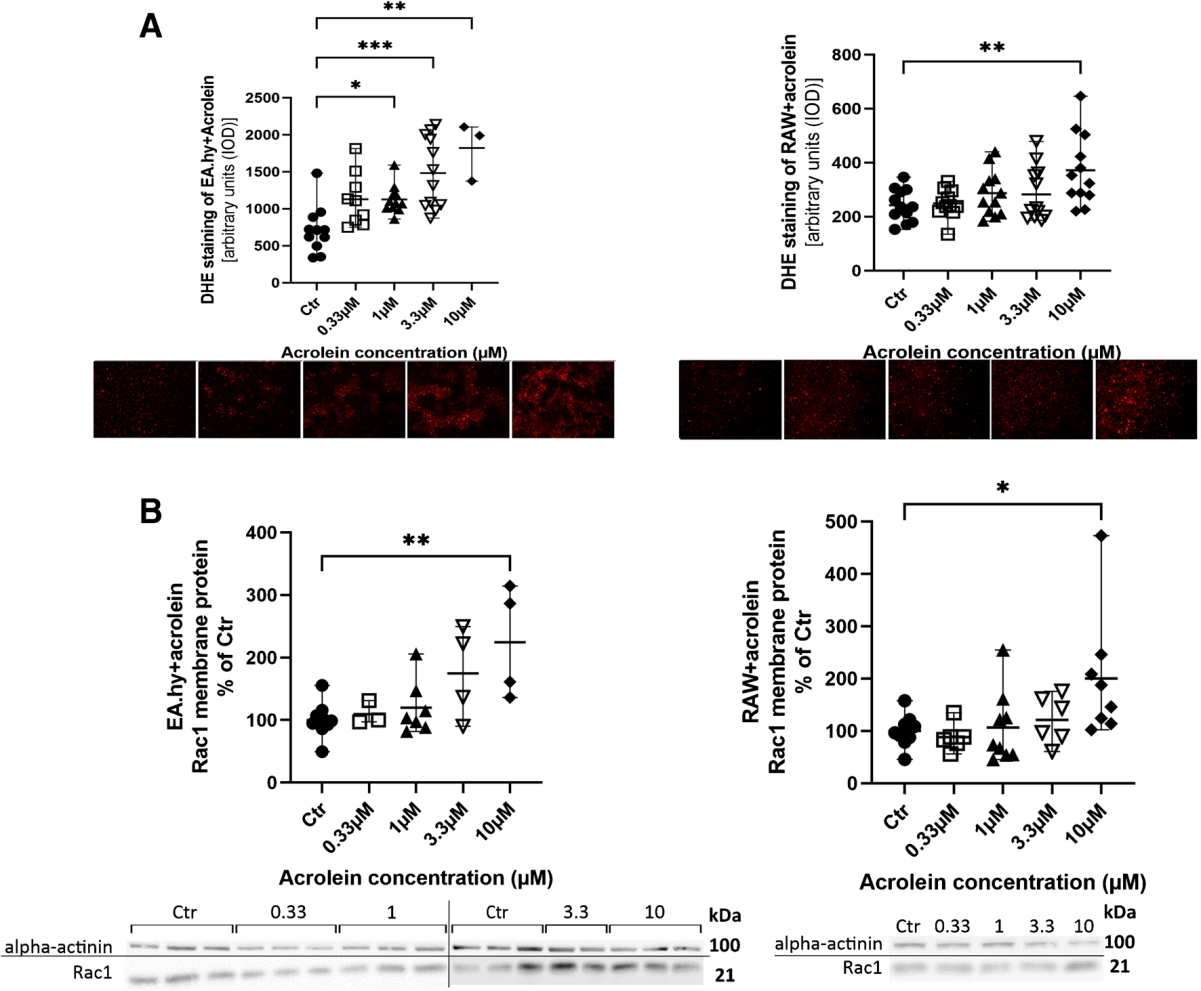
Acrolein effects on intracellular ROS formation and membrane Rac1 protein content in EA.hy and RAW cells. **A** Cells were stained with dihydroethidium (DHE) and fluorescence of the oxidation products (2-HE and E +) was recorded under a microscope (ex. 510–520 nm/em. 580–610 nm). The fluorescence intensity was quantified and shown as arbitrary units (IOD, mean ± range). Representative images are presented underneath the quantification of DHE staining. **B** Membrane protein of Rac1 after incubating the EA.hy and RAW cells with acrolein. Original blots are presented underneath the densitometric quantification. Data are presented as percentage of control (mean ± range). Each data point indicates an independent experiment. Non-parametric Kruskal–Wallis test was performed in panel **A** since the data did not pass normality test, whereas conventional 1-way ANOVA analysis was performed in panel **B**. Significance is indicated as *, **, *** and **** when *p* < 0.05, *p* < 0.01, *p* < 0.001 and *p* < 0.0001 respectively

DHE staining of EA.hy cells treated with acrolein showed trend-like increase in ROS formation with higher concentrations of acrolein. However, a statistically significant increase in ROS formation was observed in groups treated with 1 µM, 3.3 µM and 10 µM acrolein (Fig. 4A). DHE staining of RAW macrophages showed an increase in ROS formation only in cells treated with 10 µM acrolein (Fig. 4A). Localization of Rac1 to the membrane fraction of EA.hy cells increased by trend, becoming significant in the 10 µM group, which indicates activation of the NADPH oxidase complex (Fig. 4B). Localization of Rac1 to the membrane fraction of RAW macrophages showed a similar trend, reaching significance in the 10 µM treatment group (Fig. 4B).

We focused in the present study on ROS and superoxide measurements induced by acrolein as a major toxic constituent of E-cigarette vapour condensate. For E-cigarette vapour, we have shown increased ROS and superoxide formation already in vivo (by DHE fluorescence microtopography as well as HPLC analysis) in E-cigarette vapour exposed mice, even showing prevention of oxidative stress and adverse cardiovascular effects in E-cigarette vapour exposed mice with genetic deficiency of phagocytic NADPH oxidase (Nox2 knockout) [41]. In addition, we have previously shown that decreased cell viability by E-cigarette vapour condensate is improved by addition of ROS scavengers and source inhibitors such as catalase, or NADPH oxidase inhibition by the inhibitory peptide gp91 ds-tat or the synthetic drug GSK2795039.

### Acrolein effects on extracellular ROS formation in EA.hy and RAW cells measured by HPLC method

**Fig. 5:**
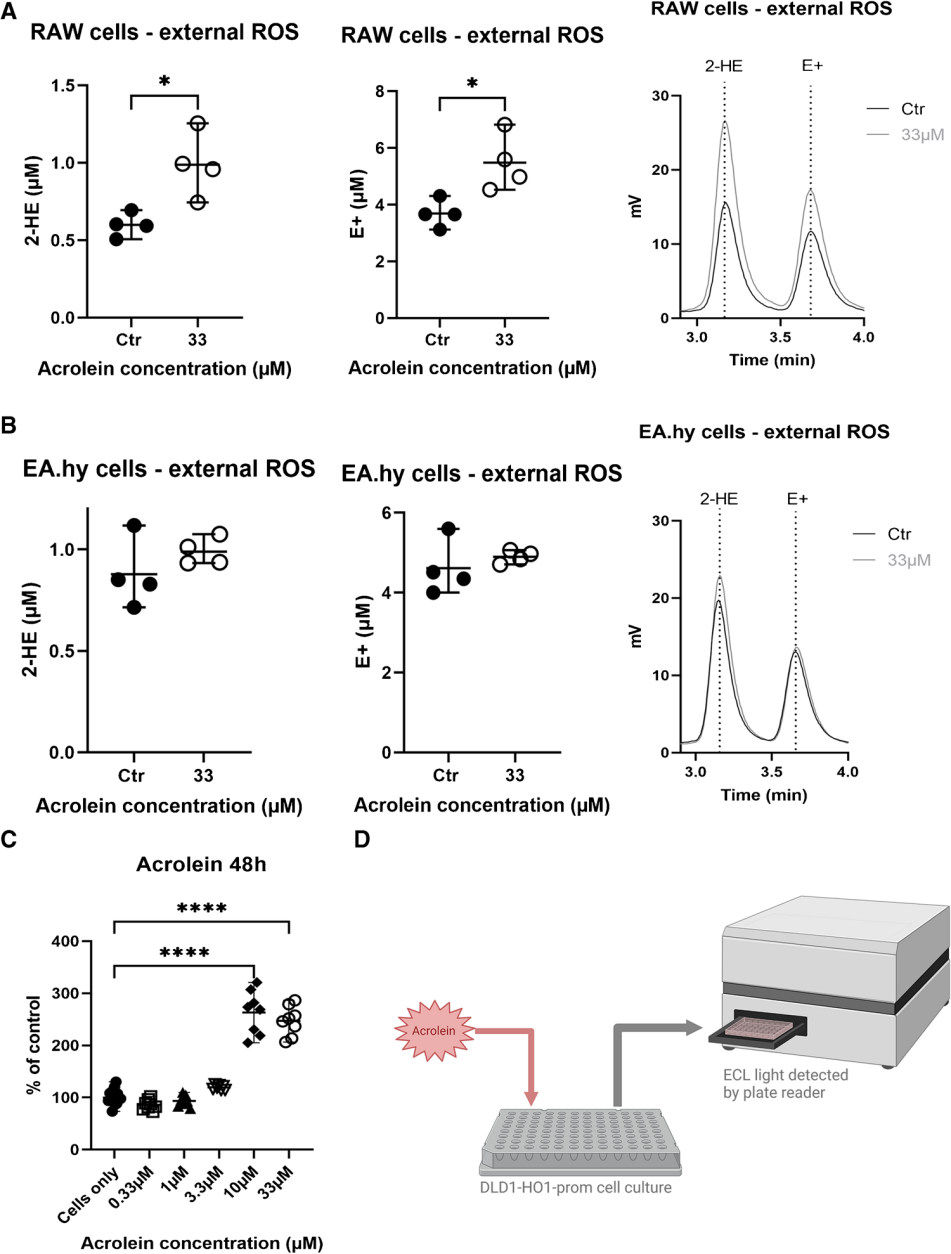
Acrolein effects on extracellular ROS formation on RAW and EA.hy cells. RAW cells (**A**) and EA.hy cells (**B**) were incubated with dihydroethidium (DHE) and the extracellular oxidation products 2-hydroxyethidium (2-HE, superoxide specific) and ethidium (E + , unspecific) in the cell culture supernatant were separated by HPLC. Specifically phagocytic NADPH oxidase produces high amounts of extracellular superoxide in immune cells. Concentrations of 2-HE and E + are shown with the representative chromatograms. Each data point indicates an independent experiment. Data are presented as absolute concentration (mean ± range). Conventional two-tailed *t*-test was used for statistical analysis. **C** Luciferase reporter assay was used to evaluate the activation of Nrf2 in acrolein-treated DLD1-HO1-prom cells. **D** The scheme summarizes the principles of the assay. Chemiluminescence after treatment with different concentrations of acrolein is shown as a percentage of control (mean ± range, *n* = 8 independent experiments). Conventional 1-way ANOVA was used for statistical analysis. Significance is indicated as *, **, *** and **** when *p* < 0.05, *p* < 0.01, *p* < 0.001 and *p* < 0.0001 respectively

Increase in extracellular ROS formation is envisaged by an increase in formation of DHE oxidation products 2-HE and E + , which was measured by HPLC with fluorescence-based detection. RAW cells showed increased extracellular ROS formation when treated with 33 µM acrolein (Fig. 5A) following the increased oxidative burst shown in Fig. 3. However, in EA.hy cells, no significant increase in extracellular ROS formation was observed, but only a minor trend (Fig. 5B). As a complementary read-out of acrolein-induced oxidative stress, the assay based on the DLD1-HO1-prom cell line showed a statistically significant increase in luciferase-dependent chemiluminescence light emission as a correlate of heme oxygenase-1 induction when treated with 10 µM and 33 µM acrolein (Fig. 5C), pointing to an activation of the Nrf2-mediated antioxidant defence.

## Discussion

With the present studies, we provide novel insights into E-cigarette-induced oxidative stress mechanisms and the importance of acrolein using in vitro models. We have previously established that short-term E-cigarette exposure causes vascular dysfunction and oxidative stress in a mouse model [41]. Here, endothelial cells and macrophages, essential for vascular homeostasis, were exposed to either E-cigarette vapour condensate or its most toxic metabolic component, acrolein. We show that both E-cigarette vapour condensate and acrolein exposure cause a concentration and time-dependent cytotoxicity that was mediated, at least partially, through oxidative stress. NADPH oxidase was identified as the primary source of ROS, as the Rac1 subunit is translocated to the membrane to form an active NADPH oxidase complex. Whereas ROS formation in response to acrolein is rather intracellular in the endothelial cells, it was mostly extracellular in the macrophages. This could be because macrophages produce most ROS extracellularly via NADPH oxidase, while sources of ROS in endothelial cells are instead located intracellularly.

E-cigarettes have previously been shown to cause oxidative stress in both in vitro and in vivo models. A study in HUVEC (human umbilical vein endothelial) cells showed that E-cigarette aerosol extract induced ROS formation, detected by a commercial fluorescence assay [2]. Another study in alveolar macrophages confirmed the presence of ROS after E-cigarette aerosol extract exposure, using a similar assay [69]. In a study by Lerner et al., it was demonstrated that E-cigarette vapour itself has high levels of ROS, which could contribute to oxidative stress in cells [44]. The study also showed that E-cigarette vapour exposure in wild-type mice caused glutathione depletion in lung tissue, pointing to a redox imbalance. Malondialdehyde (MDA), as a marker of oxidative stress, was also found to increase in both bronchoalveolar lavage fluid and lung tissue of mice exposed to E-cigarette vapour with or without nicotine and flavours for 3 days and 4 weeks [27]. Recent studies found endothelial dysfunction and vascular oxidative stress, eNOS uncoupling, and impaired nitric oxide signalling even more pronounced when E-cigarette vapour with nicotine was applied to animals [23].

The toxicity of E-cigarettes is usually attributed to nicotine and/or flavourings, since the basic components of E-cigarette liquid, propylene glycol and glycerine are considered non-toxic [25]. However, some studies have observed nicotine-independent and flavour-independent effects which mainly were attributed to aldehydes generated during the heating of E-cigarette liquid, most notably acrolein [68]. Escobar et al. demonstrated that Nrf2-mediated antioxidant defence, envisaged by NAD(P)H-quinone-dehydrogenase 1 (NQO1) and heme-oxygenase-1 (HO-1) mRNA expression, is increased even in cells treated with nicotine-free and flavour-free E-cigarette vapour [24]. The same effect was observed for acrolein in the present study, using a luciferase reporter cell assay. Total ROS levels were also increased in human bronchial epithelial cells when exposed to non-nicotine and non-flavour-containing E-cigarette vapour [64]. Our previous study showed more detrimental effects when exposing wild-type mice to non-nicotine and non-flavour-containing E-cigarette vapour [41]. In studies where propylene glycol and glycerine were individually tested as an E-cigarette liquid base, glycerine was perceived to have more pronounced effects on oxidative stress and inflammation markers [24, 64]. As acrolein is a product of glycerine degradation, this points to acrolein being an essential contributor to the observed adverse effects. The presence of acrolein was confirmed in E-cigarette vapour by different instrumental analytical methods. The relative glycerine amount in the E-cigarette liquid was strongly correlated with the acrolein concentration in the vapour [13, 22].

Acrolein is an electrophilic compound, the strongest among the α,β-unsaturated aldehydes [83]. It is known to exert its toxicity through reaction with nucleophilic residues on proteins and DNA [63], as well as through the depletion of glutathione [37]. Acrolein reacts with amino acid side chain residues with a thiol or amino group (cysteine, histidine and lysine) through Michael addition or Schiff base formation [8]. These general toxic effects are nicely reflected by the observed decreased cell viability of acrolein-treated endothelial cells and macrophages, mirrored by the cell toxicity of E-cigarette vapour condensate observed in the present study in both cell cultures.

Previous studies have shown that incubating endothelial cells with acrolein induces NADPH oxidase-dependent superoxide formation [34]. Interestingly, acrolein also inhibited NADPH oxidase activity in several studies [53, 84]. In one of the studies where acrolein was shown to inhibit NADPH oxidase-dependent oxidative burst, direct acrolein-protein adduct formation was identified with p47phox [53]. NADPH oxidase activity plays a prominent role in cardiovascular disease, as it is one of the major enzymes that catalyse the production of ROS [17, 39, 60]. Activation of NADPH oxidase was shown to increase the risk of atherosclerosis by reducing ^•^NO-mediated vasomotor function [29]. In addition, activation of the NADPH oxidase is linked to changes in the structure and function of cerebral arterioles that are associated with small vessel disease [49], and to angiotensin II-dependent activation of the enzymatic complex that increased oxidative stress and hypertension in both rats and endothelial cells [43]. Here, we demonstrate that the E-cigarette vapour condensate-mediated increase in ROS and NADPH oxidase activation is potentially regulated by acrolein, which is supported by acrolein-mediated membrane translocation of the cytosolic/regulatory NADPH oxidase subunit Rac1 as well as the primarily extracellular ROS formation (oxidative burst) in cultured macrophages.

Activator protein 1 (AP-1) and nuclear factor kappa-light-chain-enhancer of activated B cells (NF-κB) are some of the factors responsible for the transcription of NADPH oxidase subunits [47, 48]. These transcription factors are also redox-regulated, making them a target for acrolein or downstream ROS formation. AP-1 activity was shown to be increased by acrolein through activation of c-jun and activator transcription factor-2 (ATF-2) by SAPK/JNK (stress-activated protein kinases/Jun amino-terminal kinases) and p38 mitogen-activated protein kinases (p38MAPK) [61]. NF-κB and AP-1 activation might also depend on GSH/GSSG ratio, although some studies have pointed out that acrolein inhibits their activity [45, 50, 62]. There is evidence that acrolein can activate the protein kinase C (PKC), which is responsible for the phosphorylation of the cytosolic subunits of the NADPH oxidase, promoting the assembly of the activated membrane complex [33, 55, 56, 86].

Activation of the antioxidant defence through the Nrf2 pathway observed by the luciferase reporter assay might not be only due to the increase in ROS production from the activation of NADPH oxidase. Acrolein as an electrophile can induce the activation of Nrf2 independently of NADPH oxidase, by depletion of GSH [32], by limiting the enzymatic activity of thioredoxin, peroxiredoxins and other Nrf2 transcription targets, through adduct formation [70] and by potentially reacting with KEAP1 cysteine residues, promoting Nrf2-KEAP1 dissociation [31] [4]. This would imply that Nrf2-dependent antioxidant defence is activated immediately following acrolein exposure and further upon ROS production by the activated NADPH oxidase complex [72]. We speculate that acrolein and ROS-dependent activation of Nrf2 may partially compensate the oxidative damage and inflammatory reactions in response to E-cigarette vapour, preventing a more severe endothelial dysfunction and cardiovascular damage, however, without providing complete protection.

The most expressed NOX isoform in endothelial cells is NOX4, while NOX2 is highly expressed in macrophages and other phagocytic cells [1]. NOX4 does not require p47phox, p67phox and Rac1 subunits for activation [6]. Therefore, if acrolein affects NOX4, it is independent of the complex formation by promoting subunits like Rac1 to the membrane, which we observed here for the NOX2 activation. Unlike NOX2, which primarily produces superoxide radicals, NOX4 produces H_2_O_2_ which has known benefits in the cardiovascular system, such as endothelial cell growth and proliferation and regulation of endothelial barrier function [12]. H_2_O_2_ produced by NOX4 may also directly regulate endothelium-dependent vasorelaxation and eNOS expression and endothelial inflammatory signalling [7], thereby conferring cardioprotection [51]. In addition, it was shown previously that NOX4 may regulate the activation of Nrf2 during cellular redox homeostasis [65]. The NOX4-dependent protective effects are also supported by a study showing exacerbated damage by methyl vinyl ketone and acrolein, both major toxic constituents of tobacco smoke extract, in cells with impaired NOX4 signalling [80]. So far, NOX4 was not reported as a major player in E-cigarette vaping-associated pathophysiology. However, results indicate that NOX isoforms 1, 2, 4 and 5 all remain activated in smokers with end-stage chronic obstructive pulmonary disease. NOX1 and NOX4 mediate oxidative stress and inflammatory responses in response to acute cigarette smoke exposure of mice as shown using genetic deletion of the NOX isoforms. Taken together, this may suggest a role of NOX4 in E-cigarette vapour-induced organ damage [78].

Acrolein represents a metabolite of the cyclophosphamide [46] and may contribute to this anti-cancer drug’s cytostatic and cytotoxic properties. A study done in mice found that only the chemical variant of cyclophosphamide that releases both acrolein and an alkylating metabolite phosphoramide mustard, but not the one that releases only acrolein, had increased the survival time of mice injected with leukaemia cells [85], supporting only weak cytostatic effects of acrolein. In line with this previous evidence, time-dependent evaluation of cell viability in our cell culture experiments also instead supports acrolein-mediated damage to developed cells with subsequent cell death rather than cytostatic effects inhibiting mitosis and cytokinesis.

### Limitations and future improvements of the study

Future studies should check for more direct measurement of NADPH oxidase activation in endothelial cells that may complement the oxidative burst measurements in macrophages. Acrolein-protein adducts were not reported in the present study, which would open the possibility that other aldehydes in E-cigarette vapour may have contributed to the observed activation of the NADPH oxidase. In addition, different propylene glycol and glycerine ratios should be tested to provide more convincing evidence that glycerine-derived acrolein is responsible for the perceived effects.

## Conclusion

**Fig. 6:**
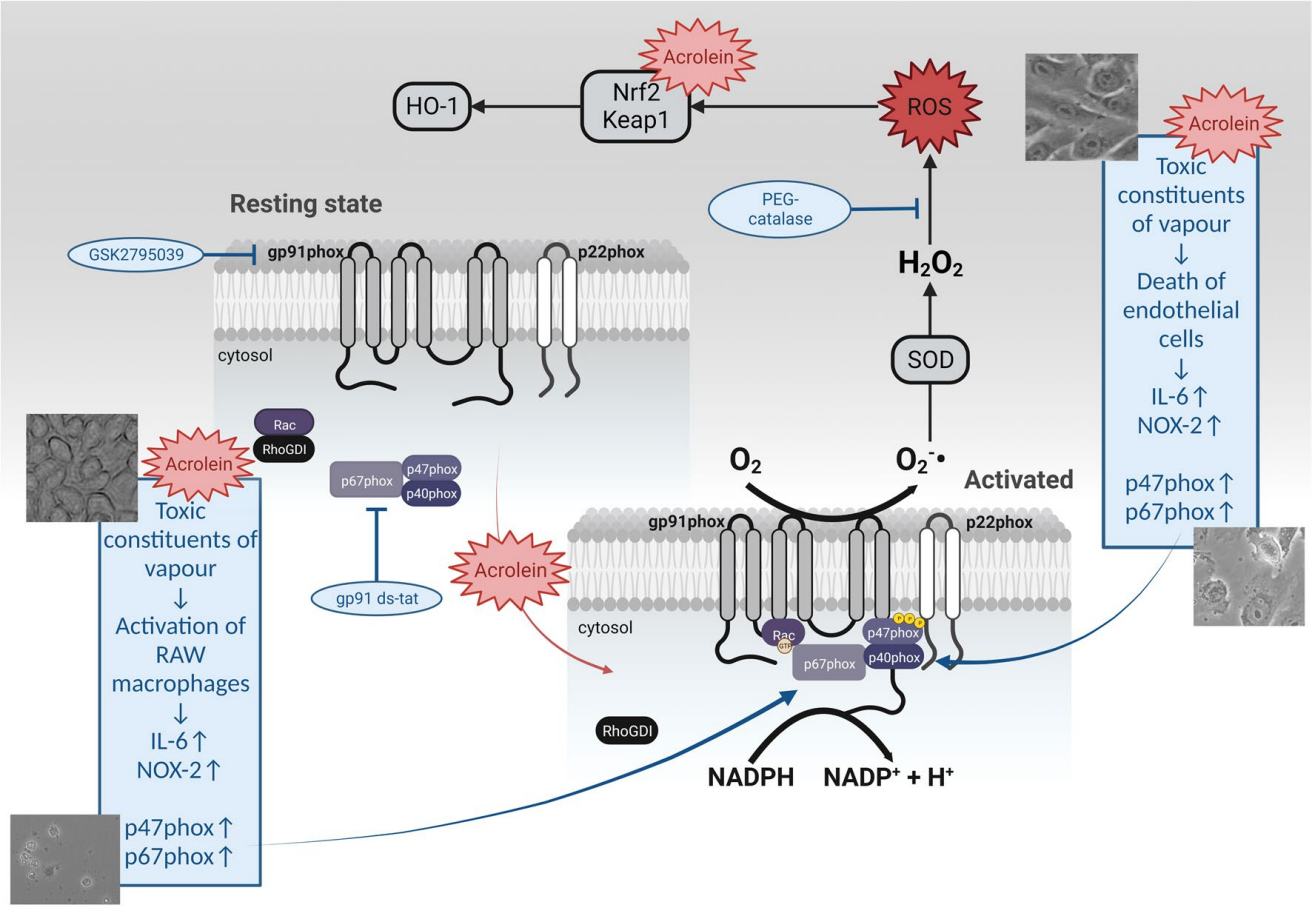
Summarizing scheme of the mechanisms involved in E-cigarette condensate and acrolein-induced oxidative stress and cell death. NADPH oxidase complex is activated via toxic constituents of vapour by translocation of subunits (p47phox, p67phox and Rac1) from cytosol to the membrane-bound gp91phox subunit (NOX2). The activated complex generates superoxide radicals, contributing to the total ROS burden. ROS activates Nrf2 transcription factor responsible for antioxidant defence, which modulates the transcription of enzymes such as heme-oxygenase-1 (HO-1). This compensatory rescue mechanism remains, however, futile. Acrolein, as an electrophile, can activate NADPH oxidase complex directly by forming adducts with cytosolic subunits or activating protein kinase C (PKC), which phosphorylates cytosolic subunits, promoting them to the membrane. In this way, acrolein increases total ROS production. Still, it can also activate Nrf2 pathway directly via electrophilic reaction with the thiols in Keap1 or indirectly via induction of ROS (redox-dependent activation of Keap1) and subsequent translocation to the nucleus. Results from our previous study [13] are shown in blue text fields. Created with BioRender.com

Here we demonstrate that E-cigarette vapour condensate has cytotoxic effects on endothelial cells and macrophages in a concentration-dependent and time-dependent manner. Cytotoxicity in cells treated with E-cigarette vapour condensate can be mimicked by treatment with acrolein. This might indicate that an appreciable part of the adverse effects of E-cigarette vapour condensate found in human endothelial cells and macrophages may arise from acrolein formed while heating E-cigarette liquid before inhaling (summarized in Fig. 6). We also showed that acrolein induces oxidative stress and triggers a compensatory antioxidant response via Nrf2-dependent induction of heme-oxygenase-1. Oxidative stress caused by acrolein is mainly based on NADPH oxidase-dependent ROS formation, as indicated by Rac1 translocation to the membrane in endothelial cells and macrophages, as well as extracellular ROS formation in macrophages in response to acrolein treatment. More research is still needed to completely understand the mechanisms linked to the toxicity of E-cigarettes, specifically those mechanisms involving E-cigarette liquid heating end products, such as acrolein.

## Data Availability

All study data are presented in the main text. Raw data and experimental materials used in this study are available upon reasonable request to the corresponding author.

## Abbreviations

*NAPDH*: Nicotinamide adenine dinucleotide phosphate
*NOX2*: Gp91phox subunit of the NADPH oxidase
*Rac1*: Ras-related C3 botulinum toxin substrate 1
*ROS*: Reactive oxygen species
